# Survival benefit and toxicity trade-offs of first-line chemoimmunotherapy in advanced gastric and gastroesophageal junction cancer: a meta-analysis of randomized trials

**DOI:** 10.3389/fonc.2026.1914121

**Published:** 2026-09-09

**Authors:** Xiaoli Zeng, Jiaxi Chen

**Affiliations:** 1 Department of Thoracic Surgery, Rehabilitation Hospital Affiliated to Nanchang University, Nanchang, Jiangxi, China; 2 Department of General Surgery, Rehabilitation Hospital Affiliated to Nanchang University, Nanchang, Jiangxi, China

**Keywords:** chemotherapy, gastric cancer, gastroesophageal junction cancer, immune checkpoint inhibitor, PD-1 inhibitor, PD-L1 inhibitor

## Abstract

**Background:**

First-line chemoimmunotherapy has become a major therapeutic strategy for advanced gastric and gastroesophageal junction cancer, yet the magnitude of survival benefit, regional consistency, and toxicity trade-offs remain important considerations. We conducted an systematic review and meta-analysis of randomized controlled trials evaluating PD-1/PD-L1 inhibitor plus chemotherapy versus chemotherapy alone or placebo plus chemotherapy.

**Methods:**

PubMed/MEDLINE, Embase, Cochrane CENTRAL, Web of Science, and ClinicalTrials.gov were searched from inception to March 1, 2026. Eligible studies enrolled adults with previously untreated unresectable, recurrent, locally advanced, or metastatic gastric or gastroesophageal junction cancer. Hazard ratios were pooled for overall survival and progression-free survival, and risk ratios were pooled for objective response rate and safety outcomes using random-effects models. Risk of bias was assessed using RoB 2, and certainty of evidence was evaluated using GRADE.

**Results:**

Seven randomized controlled trials including 6,517 patients were analyzed. PD-1/PD-L1 inhibitor plus chemotherapy significantly improved overall survival and progression-free survival. Objective response rate was also increased (RR, 1.24; 95% CI, 1.18–1.31), with negligible observed heterogeneity (I² = 0.0%). Overall survival effects were similar in global trials (HR, 0.79; 95% CI, 0.75–0.85) and Asian-only or China-only trials (HR, 0.81; 95% CI, 0.73–0.91; P for subgroup difference = 0.70). The progression-free survival effect was greater in Asian-only or China-only trials (HR, 0.66 vs. 0.78; P for subgroup difference = 0.02), although this analysis was exploratory. Combination therapy increased grade ≥3 treatment-related adverse events (RR, 1.15; 95% CI, 1.08–1.23), serious treatment-related adverse events (RR, 1.53; 95% CI, 1.29–1.83), and treatment discontinuation (RR, 1.53; 95% CI, 1.37–1.72). The pooled estimate for treatment-related death was statistically inconclusive (RR, 1.54; 95% CI, 0.75–3.16); the wide confidence interval indicated substantial imprecision and could not exclude clinically important increases or decreases in treatment-related mortality.

**Conclusions:**

First-line PD-1/PD-L1 inhibitor + chemotherapy provides a consistent survival and response benefit in advanced gastroesophageal junction or gastric cancer, but with increased clinically relevant toxicity. These findings support chemoimmunotherapy as a preferred first-line strategy for appropriately selected patients, with treatment decisions guided by biomarker status, patient fitness, and toxicity risk.

## 1 Introduction

Gastric cancer remains a global health burden and one of the leading causes of cancer-related death worldwide. In 2022, there were an estimated 969,000 new cases and 660,000 deaths from gastric cancer worldwide, highlighting the aggressive biology of this disease and the limitations of current therapeutic strategies (1). Although early diagnosis and multidisciplinary management have led to improved outcomes for selected patients with localized disease, a substantial proportion of patients present with unresectable, recurrent or metastatic gastric or gastro-esophageal junction cancer, for whom prognosis remains poor (2).

Platinum- and fluoropyrimidine-based chemotherapy has been the mainstay of first-line systemic therapy for advanced human epidermal growth factor receptor 2-negative gastric and gastro-esophageal junction adenocarcinoma for more than a decade (3). However, the survival benefit with chemotherapy alone has been modest and durable disease control is rare. These limitations have spurred intense investigation into immune checkpoint inhibition, in particular blockade of the programmed cell death protein 1/programmed death ligand 1 axis. Gastric and gastro-esophageal junction cancers are biologically heterogeneous tumors with varying immune infiltration, genomic instability, Epstein–Barr virus association in a subset of cases, microsatellite instability, and differential PD-L1 expression, all of which provide a strong biological rationale for the incorporation of immunotherapy in first-line treatment (4–6).

The first-line treatment landscape has changed substantially following the emergence of randomized evidence supporting PD-1/PD-L1 blockade combined with chemotherapy. CheckMate 649 demonstrated that nivolumab plus chemotherapy improved survival compared with chemotherapy alone, and this benefit was maintained during extended follow-up (7, 8). KEYNOTE-859 similarly showed an overall survival benefit with pembrolizumab plus chemotherapy in patients with HER2-negative advanced gastric or gastroesophageal junction adenocarcinoma (9). Subsequent phase 3 trials, including ORIENT-16, RATIONALE-305, ATTRACTION-4, and GEMSTONE-303, extended the evidence across different geographic settings, biomarker populations, chemotherapy backbones, and checkpoint inhibitors (10–13). Collectively, these findings have shifted first-line management from chemotherapy alone toward chemoimmunotherapy for appropriately selected patients (2).

But these advances have not eliminated important uncertainties. Individual trials have varied in design, enrolled populations, PD-L1 eligibility criteria, geographic distribution, chemotherapy regimens, follow-up duration, and statistical analysis populations. Some studies had all-comer populations, while others required or emphasized PD-L1-enriched subgroups. Moreover, the degree of benefit varied among trials, with some yielding clear improvements in overall survival and progression-free survival and others demonstrating more pronounced effects on progression-free survival than on overall survival. These differences are clinically relevant. Treatment decisions in advanced gastric and gastroesophageal junction cancer require clinicians to balance the expected survival benefit against toxicity, treatment discontinuation, patient fitness, biomarker status, and regional practice patterns (7–14).

Safety is also important when interpreting the clinical value of first-line chemoimmunotherapy. Combining PD-1/PD-L1 inhibitors with cytotoxic chemotherapy may increase grade ≥3 treatment-related adverse events, serious treatment-related adverse events, and treatment discontinuation. Although immune-related adverse events are generally manageable, some may be serious, persistent, or fatal. They may also overlap with chemotherapy-related toxicities in routine clinical practice. A balanced synthesis of efficacy and safety outcomes is therefore needed to assess the net clinical value of first-line chemoimmunotherapy.

The role of first-line immunotherapy-based combinations in advanced gastric and gastro-esophageal junction cancer has been supported by previous meta-analyses but the evidence base continues to evolve (13, 15, 16). However, with more mature follow-up from pivotal trials, and publication of further randomized controlled trials, a more complete assessment of treatment effects across survival, response, and toxicity outcomes is now possible. This update to the systematic review is especially important because the field has moved from early proof-of-concept evidence to a broader question of how consistently chemoimmunotherapy improves clinically meaningful outcomes across diverse trial settings.

Consequently, we performed a comprehensive review and meta-analysis of randomized controlled trials comparing first-line PD-1/PD-L1 inhibitors combined with chemotherapy against treatment or placebo combined with chemotherapy in patients with advanced gastric or gastro-esophageal junction cancer. The primary endpoint was the impact of combination treatment on overall survival and progression-free survival. The secondary objectives encompassed the evaluation of the objective response rate and significant safety outcomes, including grade 3 or higher treatment-related adverse events, serious treatment-related adverse events, treatment discontinuation due to adverse events, and treatment-related mortality. We also examined whether the treatment results were comparable in the global and Asian or China-specific trial populations.

## 2 Materials and methods

### 2.1 Study design and reporting framework

In patients with advanced gastric or gastro-esophageal junction cancer, this systematic review and meta-analysis was carried out to assess the safety and effectiveness of first-line programmed cell death protein 1/programmed death ligand 1 (PD-1/PD-L1) inhibitors in combination with chemotherapy as opposed to chemotherapy alone or placebo plus chemotherapy. The Preferred Reporting Items for Systematic Reviews and Meta-Analyses 2020 standards were followed in the planning and reporting of the review. The Population, Intervention, Comparator, Outcomes, and Study design framework was used to structure the review question. The review was not prospectively registered in PROSPERO or another systematic review registry. However, the review question, eligibility criteria, primary and secondary outcomes, and analytical framework were established before data extraction and quantitative synthesis and were applied consistently throughout the review. These included overall survival and progression-free survival as the primary outcomes, objective response rate and prespecified safety outcomes as secondary outcomes, and the planned random-effects meta-analytic approach. The review was conducted and reported in accordance with the PRISMA 2020 statement.

### 2.2 Eligibility criteria

The randomized controlled trials that enrolled adults with previously untreated, unresectable, locally advanced, recurrent, or metastatic gastric cancer or gastro-esophageal junction adenocarcinoma were included. Eligible trials had to evaluate a PD-1 or PD-L1 inhibitor plus first-line systemic chemotherapy and compare the regimen with chemotherapy alone or placebo plus chemotherapy. Trials were required to report at least one pre-specified efficacy or safety outcome.

Overall survival and progression-free survival were the primary results. The objective response rate was one of the secondary efficacy goals. Safety outcomes included grade ≥3 treatment-related adverse events (TRAEs), serious TRAEs, treatment discontinuation due to adverse events (AEs) or TRAEs, and treatment-related death.

Studies were excluded if they were non-randomized, single-arm, observational, retrospective, phase 1 dose-escalation studies, reviews, editorials, case reports or pooled *post hoc* analyses without an independent randomized comparison. Exclusion criteria were trials evaluating immune checkpoint inhibitor monotherapy, maintenance immunotherapy after chemotherapy, perioperative or neoadjuvant therapy in resectable disease, second- or later-line therapy, HER2-positive regimens in which both arms contained anti-HER2 therapy, dual-checkpoint blockade, bispecific immunotherapy or any regimen that did not isolate the effect of a PD-1 or PD-L1 inhibitor + chemotherapy compared with chemotherapy alone or placebo + chemotherapy.

### 2.3 Information sources and literature search

The systematic review and meta-analysis adhered to the PRISMA 2020 recommendations. We conducted a search of PubMed/MEDLINE, Embase, Cochrane CENTRAL, and Web of Science from their establishment to 1^st^ March 2026. We conducted a search on ClinicalTrials.gov for ongoing, completed, or unpublished randomized trials. A manual examination of the reference lists from the included studies and pertinent reviews was performed to discover further studies.

The search strategy combined terms related to gastric or gastroesophageal junction cancer, chemotherapy, PD-1/PD-L1 immune checkpoint inhibitors, and randomized trials. Search terms included combinations of: “gastric cancer,” “stomach cancer,” “gastric adenocarcinoma,” “gastroesophageal junction,” “GEJ,” “advanced,” “metastatic,” “unresectable,” “programmed death ligand 1,” “PD-1,” “programmed death 1,” “PD-L1,” “nivolumab,” “pembrolizumab,” “sintilimab,” “tislelizumab,” “sugemalimab,” “chemotherapy,” “capecitabine,” “oxaliplatin,” “fluoropyrimidine,” “cisplatin,” “FOLFOX,” “XELOX,” “CAPOX,” “randomized,” “randomized,” “phase 3,” and “phase III.” No language restrictions were applied at the search stage. Non-English studies were considered eligible if sufficient data were available for extraction.

### 2.4 Study selection

All records obtained via database and registry searches were imported into Zotero. Duplicate records were detected and eliminated before screening. Two reviewers separately evaluated titles and abstracts against predetermined inclusion criteria. Records deemed possibly eligible by either review author were obtained for comprehensive evaluation.

The identical two reviewers independently assessed the eligibility of the complete papers. Disagreements at the level of title/abstract or full-text were resolved by discussion; those that could not be resolved were decided by a third reviewer. All excluded full-text articles were recorded with reasons for exclusion and summarized in accordance with PRISMA 2020 guidance.

### 2.5 Data extraction

Data were separately retrieved by two reviewers using a standardized extraction form. Characteristics of the included studies were extracted including trial name, first author, year of publication, study phase, blinding, geographic region, number of centers, eligibility criteria, cancer site, HER2 status, PD-L1 eligibility or analysis population, sample size, intervention and comparator regimens, chemotherapy backbone, duration of follow-up, and primary endpoints.

Hazard ratios with corresponding 95% confidence intervals were obtained for overall survival and progression-free survival in time-to-event outcomes. For the primary meta-analysis, intention-to-treat or all-randomized estimates were used whenever available. If a trial prospectively enrolled only a biomarker-selected population, the estimate from that randomized eligible population was used because no corresponding all-comer population existed. Thus, KEYNOTE-062 contributed data from patients with PD-L1 CPS ≥1, and GEMSTONE-303 contributed data from patients with PD-L1 CPS ≥5. In trials that enrolled all-comer populations and additionally reported PD-L1-enriched analyses, the biomarker-enriched estimates were not combined with the intention-to-treat estimates in the primary meta-analysis. This approach avoided including multiple or overlapping estimates from the same trial. Combined positive score (CPS)- and tumor area positivity (TAP)-based estimates were not treated as interchangeable because the assays, scoring systems, and thresholds differed across studies.

For binary outcomes, including objective response rate and safety endpoints, the number of patients experiencing the event and the total number analyzed in each treatment group were extracted from the primary publication, **Supplementary Materials**, and other eligible trial reports. When exact event counts were not reported but arm-specific percentages and corresponding denominators were available for the relevant randomized analysis population, approximate event counts were calculated and recorded as such. A trial was included in a binary-outcome meta-analysis only when sufficient arm-level data were available to calculate a risk ratio. Data were not reconstructed when the relevant denominator was unclear, when percentages referred to a biomarker subgroup or response-evaluable population that could not be matched across treatment arms, or when rounding could lead to uncertain event counts. Outcomes for which treatment-arm event counts could not be extracted or reliably reconstructed were treated as missing and were not imputed. For treatment discontinuation, the definition reported by each individual trial was retained. Because reporting was not fully uniform across studies, discontinuation due to adverse events and discontinuation due to treatment-related adverse events were treated as closely related safety outcomes for the pooled analysis, while the difference in attribution was considered when interpreting the result.

If more than one publication or report existed for the same trial, the most complete or mature dataset was chosen for each outcome. If survival and safety outcomes were reported in different publications, the source used for each outcome was recorded separately. One value for each meta-analysis was provided for each trial.

### 2.6 Risk of bias assessment

Two reviewers assessed risk of bias independently using the revised Cochrane Risk of Bias tool for randomized trials, version 2.0. Assessments were done at the outcome level for the primary efficacy outcomes and summarized at the study level for reporting. The subsequent domains of RoB 2 were evaluated: bias arising from the randomization process, bias due to deviations from intended interventions, bias from absent outcome data, bias in outcome assessment, and bias in the selection of reported results.

Each domain was assessed as having low risk of bias, some concerns, or high risk of bias in accordance with RoB 2 guidelines. Overall risk of bias evaluations for each study were determined by domain-level assessments. Discrepancies among reviewers were reconciled through conversation; any unresolved conflicts were adjudicated by a third reviewer.

Particular attention was given to allocation concealment, blinding, protocol-specified outcome reporting, completeness of follow-up, and use of blinded independent central review for progression-free survival and objective response outcomes. Blinding was not thought to be likely to bias overall survival, an objective endpoint, versus investigator-assessed progression or response outcomes. Traffic-light and summary plots were used to present risk-of-bias judgments.

### 2.7 Certainty of evidence

The Grading of Recommendations Assessment, Development and Evaluation (GRADE) approach was used to assess certainty of evidence for the principal outcomes. Randomized evidence was initially considered high certainty and was evaluated for potential downgrading across the domains of risk of bias, inconsistency, indirectness, imprecision, and publication bias. Clinical heterogeneity across trials, including differences in geographic setting, biomarker eligibility, chemotherapy backbone, and checkpoint inhibitor, was considered in the assessment of indirectness and inconsistency. Downgrading for inconsistency was based on the magnitude and direction of study-specific effects together with I² and τ² rather than on clinical heterogeneity alone. Risk-of-bias judgments were considered in relation to the contribution of studies with domain-specific concerns and the robustness of pooled estimates in sensitivity analyses.

### 2.8 Statistical analysis

The meta-analysis was conducted using aggregate trial-level data. Hazard ratios (HRs) with corresponding 95% confidence intervals (CIs) were used as the effect measure for time-to-event outcomes, including overall survival and progression-free survival. Risk ratios (RRs) with 95% CIs were used for binary outcomes, including objective response rate and safety outcomes. Effect estimates were transformed to the natural logarithmic scale, and their standard errors were derived from the reported 95% CIs before pooling. Pooled estimates were subsequently back-transformed for presentation.

Random-effects models were used for all primary meta-analyses because clinical and methodological heterogeneity was anticipated across trials, including differences in geographic setting, biomarker eligibility, chemotherapy backbone, immune checkpoint inhibitor, blinding, follow-up duration, and outcome definitions. The primary analyses used inverse-variance weighting with the DerSimonian–Laird estimator of between-study variance. The regional subgroup estimates were calculated using the same random-effects model, and differences between subgroups were evaluated using Cochran’s Q test for subgroup differences. Fixed-effect models and Hartung–Knapp-adjusted random-effects models were used only as sensitivity analyses and not as the primary analytical approach. The Hartung–Knapp method was applied where the available number of studies and outcome data permitted estimation, to assess whether accounting for uncertainty associated with a small number of trials materially altered the precision or statistical interpretation of the pooled estimates.

For overall survival and progression-free survival, an HR below 1.0 favored PD-1/PD-L1 inhibitor plus chemotherapy. For objective response rate, an RR above 1.0 favored combination therapy. For adverse-event outcomes, an RR above 1.0 indicated a higher risk of toxicity with PD-1/PD-L1 inhibitor plus chemotherapy.

Statistical heterogeneity was assessed using Cochran’s Q test, the between-study variance estimate (τ²), and the I² statistic. I² values of approximately 25%, 50%, and 75% were interpreted as indicating low, moderate, and substantial observed heterogeneity, respectively. Because I² is an estimate and may be imprecise when only a small number of studies are available, it was interpreted together with the magnitude, direction, and precision of the study-specific effect estimates. An I² value of 0% was described as negligible observed heterogeneity rather than evidence of complete homogeneity.

Prespecified trial-level subgroup analyses compared global or multinational trials with Asian-only or China-only trials. Subgroup estimates were calculated using the same random-effects framework as the primary analyses. Differences between subgroups were evaluated using Cochran’s Q test for subgroup differences, and the corresponding P values for interaction were reported. Because each subgroup contained only three or four trials, these tests had limited power and were considered exploratory. Moreover, global trials included participants from several geographic regions, including Asia; therefore, the analysis compared trial settings rather than individual-level geographic or ethnic groups. Subgroup findings were interpreted cautiously and were not considered evidence of patient-level effect modification.

An additional exploratory analysis by immune checkpoint target was considered; however, a formal meta-analysis according to PD-L1 selection status was not performed. Only two trials prospectively restricted enrollment according to PD-L1 expression, and these trials used different CPS thresholds. Other trials enrolled all-comer populations but reported subgroup results using varying CPS or TAP definitions. A trial-level comparison between biomarker-selected and all-comer studies would therefore have been underpowered and vulnerable to ecological bias, because differences between trials could not be attributed specifically to PD-L1 status. For clinical context, trial-reported overall survival and progression-free survival hazard ratios across available PD-L1-defined populations and thresholds were additionally extracted and summarized descriptively, these estimates were not quantitatively pooled.

Robustness was evaluated using leave-one-out sensitivity analyses, in which each study was sequentially excluded and the pooled estimate recalculated. Fixed-effect models were also examined as an alternative analytical approach. Where estimable, Hartung–Knapp-adjusted random-effects analyses were performed to assess the effect of small-study uncertainty on the precision of pooled estimates. These sensitivity analyses were interpreted in relation to the direction, magnitude, and statistical significance of the primary findings.

Funnel plots were visually inspected for overall survival and progression-free survival to explore potential publication bias and small-study effects. Formal tests of funnel-plot asymmetry were not performed because fewer than 10 studies contributed to these outcomes, resulting in limited statistical power and potentially unreliable estimates. All analyses were conducted using R version 4.4.1 with the meta and metafor packages.

## 3 Results

### 3.1 Study selection

The systematic search yielded 968 records from electronic databases and trial registries. After deduplication, 772 records were screened based on title and abstract. Of these, 751 records were excluded and 21 full-text articles assessed for eligibility.

Seven independent randomized controlled trials met the pre-specified eligibility criteria and were included in the primary meta-analysis: KEYNOTE-062, CheckMate 649, KEYNOTE-859, RATIONALE-305, ORIENT-16, ATTRACTION-4, and GEMSTONE-303. The most mature eligible follow-up data available within the search period were used for each outcome, including the 5-year follow-up of CheckMate 649 and the long-term follow-up of ATTRACTION-4. Each trial was included once in each meta-analysis.

**Figure 1:**
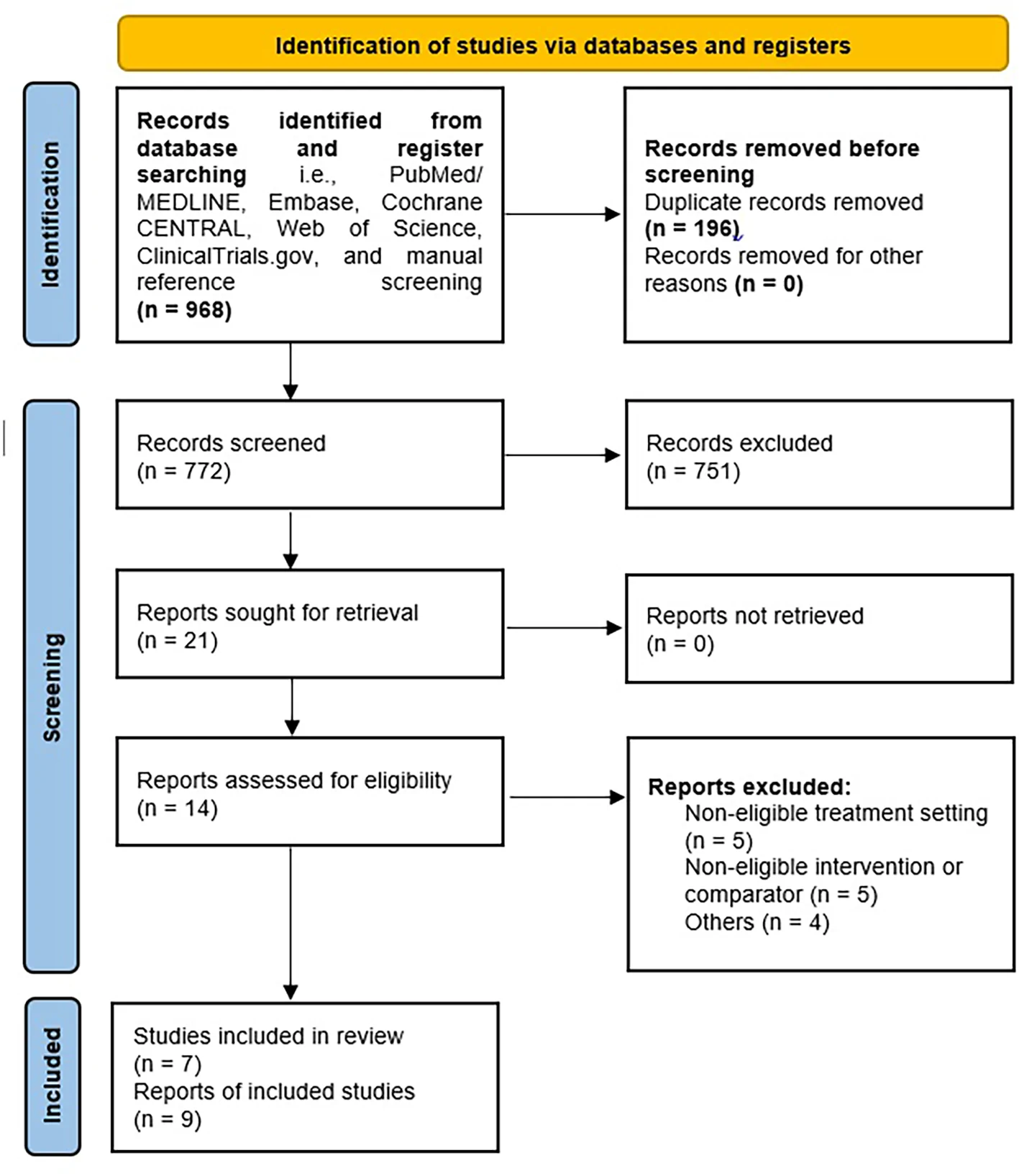
PRISMA 2020 flow diagram of study identification, screening, eligibility assessment, and inclusion.

Excluded studies from the main analysis included trials with dual-checkpoint blockade, maintenance immunotherapy, HER2-positive regimens with anti-HER2 therapy in both arms, perioperative or neoadjuvant treatment, and second-line or later-line therapy. The study-selection process is summarized in **Figure 1**.

### 3.2 Characteristics of included studies

The seven trials included in the analysis enrolled 6,517 randomized patients in the comparison arms relevant to this meta-analysis, including 3,267 patients assigned to PD-1/PD-L1 inhibitor + chemotherapy and 3,250 patients assigned to chemotherapy alone or placebo + chemotherapy. The principal characteristics of the included trials are summarized in **Table 1**. Additional details regarding study design, eligibility criteria, treatment regimens, and biomarker-defined analysis populations are provided in **Supplementary Table 1**.

**Table 1:** Principal characteristics of the included randomized controlled trials.

Trial	Region	Design	Population/biomarker eligibility	Experimental treatment	Comparator	Sample size, n	Primary endpoint(s)
CheckMate 649	Global	Open-label phase 3	Untreated HER2-negative advanced gastric, GEJ, or esophageal adenocarcinoma; enrolled regardless of PD-L1 expression; primary analysis in CPS ≥5	Nivolumab plus XELOX or FOLFOX	XELOX or FOLFOX	789/792	OS and PFS in CPS ≥5
KEYNOTE-062	Global	Randomized phase 3; partially blinded	Untreated advanced gastric or GEJ cancer with PD-L1 CPS ≥1	Pembrolizumab plus cisplatin and fluorouracil or capecitabine	Placebo plus chemotherapy	257/250	OS and PFS in CPS ≥1 and CPS ≥10
KEYNOTE-859	Global	Double-blind, placebo-controlled phase 3	Untreated HER2-negative advanced gastric or GEJ adenocarcinoma; all-comer population with CPS-defined analyses	Pembrolizumab plus FP/cisplatin or CAPOX	Placebo plus the same chemotherapy	790/789	OS
ORIENT-16	China	Double-blind, placebo-controlled phase 3	Untreated advanced gastric or GEJ adenocarcinoma; all-comer population with CPS ≥5 subgroup	Sintilimab plus XELOX	Placebo plus XELOX	327/323	OS in all randomized patients and CPS ≥5
RATIONALE-305	Global	Double-blind, placebo-controlled phase 3	Untreated HER2-negative advanced gastric or GEJ adenocarcinoma; all-comer and TAP ≥5% populations	Tislelizumab plus CAPOX or FP/cisplatin	Placebo plus the same chemotherapy	501/496	OS in TAP ≥5% and all randomized patients
ATTRACTION-4	East Asia	Double-blind, placebo-controlled phase 2/3	Untreated HER2-negative advanced or recurrent gastric or GEJ cancer; enrolled regardless of PD-L1 expression	Nivolumab plus SOX or CAPOX	Placebo plus SOX or CAPOX	362/362	Centrally assessed PFS and OS
GEMSTONE-303	China	Double-blind, placebo-controlled phase 3	Untreated advanced gastric or GEJ adenocarcinoma with PD-L1 CPS ≥5	Sugemalimab plus CAPOX	Placebo plus CAPOX	241/238	OS and investigator-assessed PFS

CAPOX, capecitabine plus oxaliplatin; CPS, combined positive score; FOLFOX, fluorouracil, leucovorin, and oxaliplatin; FP, fluoropyrimidine; GEJ, gastroesophageal junction; OS, overall survival; PD-L1, programmed death ligand 1; PFS, progression-free survival; SOX, S-1 plus oxaliplatin; TAP, tumor area positivity; XELOX, capecitabine plus oxaliplatin. Sample sizes are presented as experimental/control.

Six trials tested anti-PD-1 antibodies: nivolumab (ATTRACTION-4 & CheckMate 649), pembrolizumab (KEYNOTE-062 and KEYNOTE-859), sintilimab (ORIENT-16), and tislelizumab (RATIONALE-305). One study evaluated an anti-PD-L1 antibody, sugemalimab (GEMSTONE-303).

The majority of trials included patients with unresectable, locally progressed, recurrent, or metastatic gastric or gastro-esophageal junction adenocarcinoma that had not received prior treatment. Patients with esophageal adenocarcinoma were also included in CheckMate 649. Chemotherapy regimens were based on fluoropyrimidine and platinum, as per individual trial protocols. The comparators were chemotherapy alone, or placebo with the same chemotherapy regimen.

Four trials were global/multinational studies: KEYNOTE-062, KEYNOTE-859, CheckMate 649, and RATIONALE-305. Three trials were Asian-only or China-only studies: ATTRACTION-4, ORIENT-16 and GEMSTONE-303. Six trials were phase 3 studies. ATTRACTION-4 was a randomized phase 2/3 study. Most trials were placebo-controlled and double-blind; CheckMate 649 was an open-label trial. PD-L1 eligibility and analysis populations varied substantially across trials. KEYNOTE-062 required CPS ≥1, whereas GEMSTONE-303 required CPS ≥5. CheckMate 649, KEYNOTE-859, ORIENT-16, RATIONALE-305, and ATTRACTION-4 enrolled broader or all-comer populations, although several reported additional biomarker-enriched analyses using CPS or TAP thresholds. For the primary meta-analysis, all-randomized estimates were used when available; biomarker-enriched subgroup estimates from all-comer trials were not substituted for or pooled together with the corresponding intention-to-treat estimates. For clinical reference, the trial-reported OS and PFS hazard ratios across available PD-L1-defined populations and thresholds are summarized in **Supplementary Table 2**.

### 3.3 Risk of bias assessment

**Figure 2:**
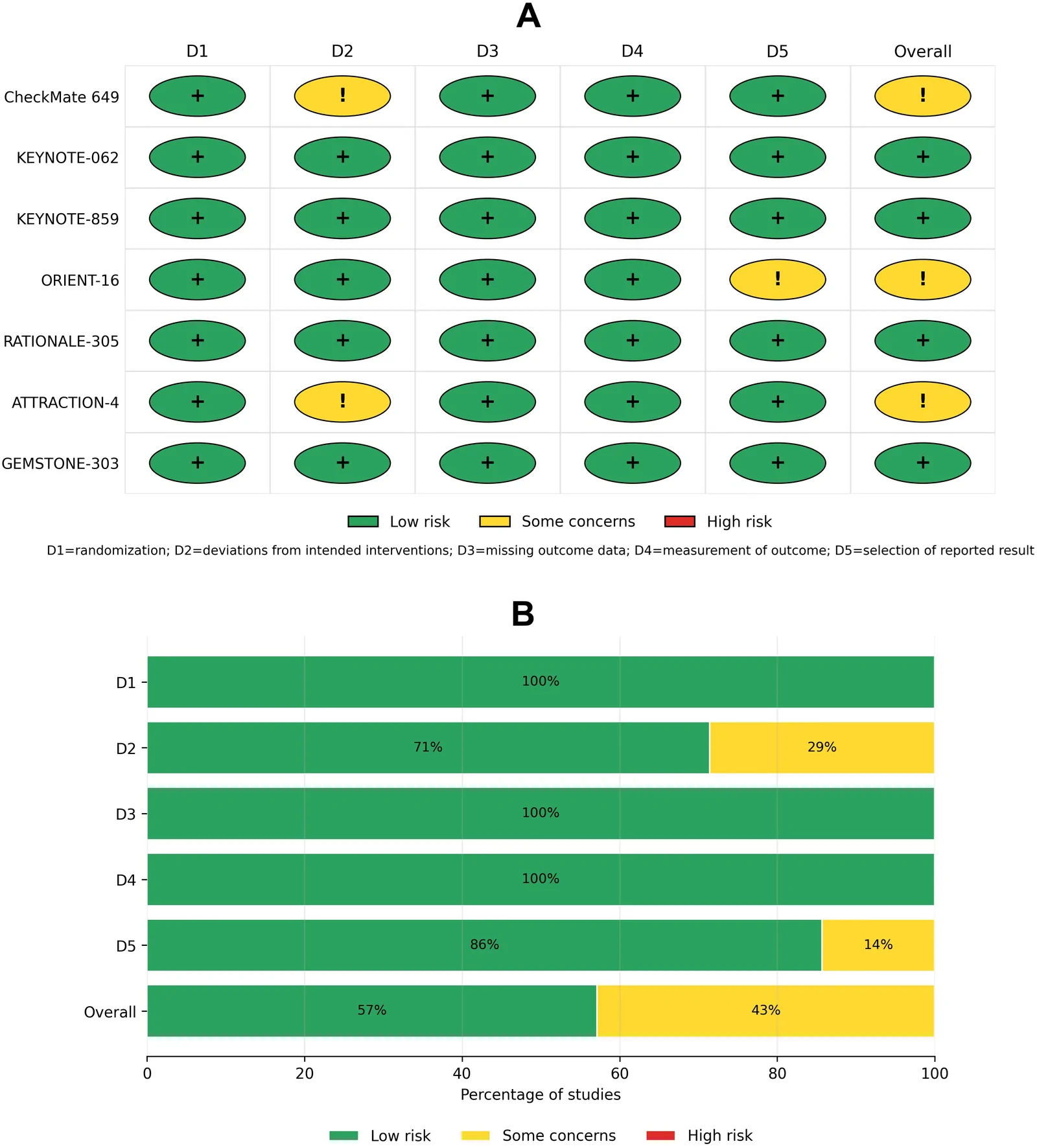
Risk of bias assessment of included randomized controlled trials. **(A)** Traffic-light plot showing study-level risk-of-bias judgments across the five RoB 2 domains and overall risk of bias. **(B)** Summary plot showing the percentage of included studies judged as low risk, some concerns, or high risk of bias across each RoB 2 domain and overall.

The risk-of-bias assessments are presented in **Figures 2A, B**. Four trials—KEYNOTE-062, KEYNOTE-859, RATIONALE-305, and GEMSTONE-303—were judged to have a low overall risk of bias. Three trials—CheckMate 649, ORIENT-16, and ATTRACTION-4—were judged to have some concerns. No trial was classified as having a high overall risk of bias.

For CheckMate 649, some concerns arose primarily in the domain of deviations from intended interventions because of the open-label design. ORIENT-16 was judged to have some concerns in the selection of the reported result because of uncertainty regarding the timing and specification of the primary analyses. ATTRACTION-4 had some concerns related to deviations from intended interventions, including the potential influence of post-protocol therapy on the interpretation of overall survival. The remaining assessed domains were generally judged to be at low risk, and missing outcome data were not considered an important source of bias across the included trials.

### 3.4 Meta-analytical results

The pooled efficacy and safety estimates are summarized in **Table 2**.

**Table 2:** Summary of pooled meta-analysis results and GRADE certainty of evidence.

Pooled meta-analysis Results							GRADE certainty of evidence	
Outcome	No. of studies	Effect measure	Pooled effect	95% CI	I²	Interpretation	Certainty of evidence	Main reason for judgment
Overall survival	7	HR	0.80	0.76–0.84	0.0%	Significant OS benefit	High	Randomized evidence directly addressing the review question; narrow confidence interval excluding the null; consistent direction of effect with negligible observed statistical heterogeneity; no trial was judged at high overall risk of bias. Clinical heterogeneity was not considered sufficient to warrant downgrading because the treatment effect remained consistent across trials and sensitivity analyses.
Progression-free survival	7	HR	0.75	0.70–0.80	22.9%	Significant PFS benefit	High	Randomized evidence directly addressing the review question; narrow confidence interval excluding the null; consistent direction of effect with low observed statistical heterogeneity; no trial was judged at high overall risk of bias. Although clinical and methodological differences were present across trials, they did not produce important inconsistency in the pooled PFS effect.
Objective response rate	6	RR	1.24	1.18–1.31	0.0%	Higher response rate	Moderate	RATIONALE-305 lacked sufficient arm-level responder data; response assessment varied across trials, and some risk-of-bias concerns were present.
Grade ≥3 TRAEs	7	RR	1.15	1.08–1.23	51.9%	Increased severe treatment-related toxicity	Moderate	Moderate heterogeneity and variation in safety reporting across trials.
Serious TRAEs	5	RR	1.53	1.29–1.83	55.9%	Increased serious treatment-related toxicity	Moderate	Moderate heterogeneity and fewer contributing studies.
Discontinuation due to AEs/TRAEs	6	RR	1.53	1.37–1.72	12.4%	Increased discontinuation	Moderate	Variation in trial-level definitions of discontinuation due to AEs versus TRAEs, limiting complete comparability across studies.
Treatment-related death	7	RR	1.54	0.75–3.16	48.0%	Statistically inconclusive; clinically important harm or benefit cannot be excluded	Low	Rare events and a wide confidence interval that included clinically important increases and decreases in mortality risk.

AE, adverse event; CI, confidence interval; GRADE, Grading of Recommendations Assessment, Development and Evaluation; HR, hazard ratio; k, number of trials; OS, overall survival; PFS, progression-free survival; RR, risk ratio; TRAE, treatment-related adverse event.

#### 3.4.1 Efficacy outcomes

##### 3.4.1.1 Overall survival

**Figure 3:**
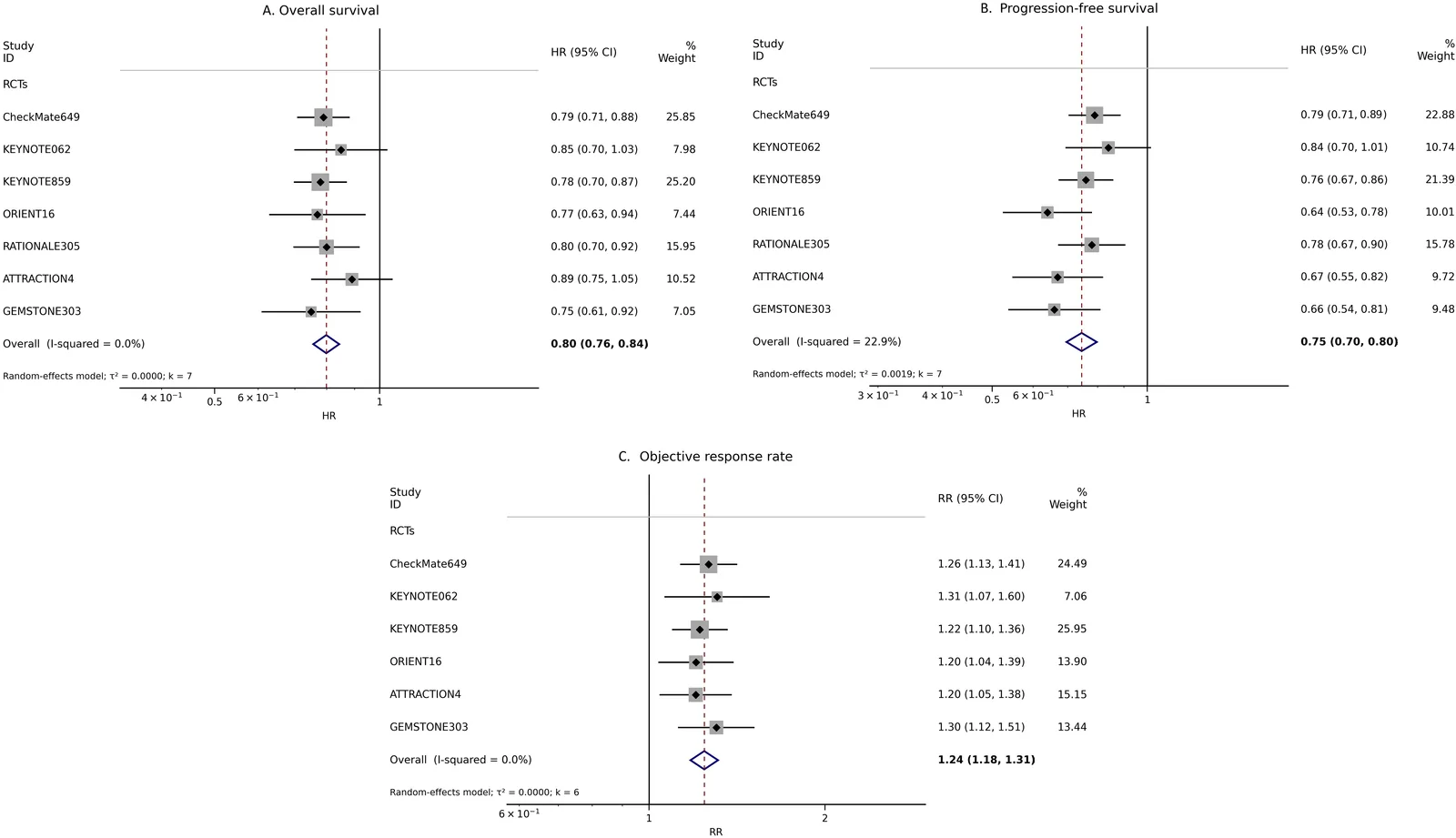
Forest plots of pooled efficacy outcomes. Forest plots comparing first-line PD-1/PD-L1 inhibitor plus chemotherapy with chemotherapy alone or placebo plus chemotherapy for **(A)** overall survival, **(B)** progression-free survival, and **(C)** objective response rate.

Data from all seven trials were available for the overall survival analysis. **Figure 3A** shows the forest plot. The use of PD-1/PD-L1 inhibitors with chemotherapy demonstrated markedly improved overall survival compared to chemotherapy alone or placebo combined with chemotherapy. The pooled hazard ratio was 0.80 (95% CI, 0.76–0.84), indicating an approximately 20% lower hazard of death. The point estimate indicated negligible observed between-study heterogeneity (I² = 0.0%). However, this estimate should be interpreted cautiously because only seven trials contributed to the analysis. The direction of the survival benefit was consistent across the included studies. KEYNOTE-859, CheckMate 649, RATIONALE-305, ORIENT-16, and GEMSTONE-303 all showed significant overall survival improvement with the addition of PD-1/PD-L1 blockade to chemotherapy. In the KEYNOTE-062 study, pembrolizumab plus chemotherapy did not demonstrate superiority to chemotherapy for overall survival in the populations studied.

##### 3.4.1.2 Progression-free survival

Data from all seven trials were incorporated into the progression-free survival analysis. **Figure 3B** displays the forest plot. The combination of PD-1/PD-L1 inhibitors with chemotherapy demonstrated a significantly superior progression-free survival compared to chemotherapy alone or placebo combined with chemotherapy. The pooled hazard ratio was 0.75 (95% CI, 0.70–0.80), which means that there was an approximately 25% lower hazard of progression or death. Between-study heterogeneity was low (I^2^ = 22.9%). The size of the progression-free survival benefit differed among the trials, but consistently favored the chemoimmunotherapy combination.

##### 3.4.1.3 Objective response rate

Six trials contributed to the pooled objective response rate analysis. RATIONALE-305 was not included because re-examination of the full publication and available **Supplementary Materials** did not identify treatment-arm responder counts, or arm-specific response percentages with clearly corresponding denominators, for the randomized population used in this meta-analysis. Consequently, an ORR risk ratio could not be extracted or reliably reconstructed without making assumptions regarding the analysis population or rounded event counts. Consistent with the prespecified data-handling approach, the missing outcome was not imputed. The exclusion was limited to the ORR analysis; RATIONALE-305 remained included in all other analyses for which sufficient data were available. **Figure 3C** shows the forest plot. The combination of PD-1/PD-L1 inhibitor and chemotherapy was associated with a higher objective response rate compared with chemotherapy alone or placebo with chemotherapy. The pooled risk ratio was 1.24 (95% CI, 1.18–1.31) with negligible observed between-study heterogeneity (I^2^ = 0.0%). This finding implies that the survival benefit of PD-1/PD-L1 inhibitor plus chemotherapy was related to a higher radiographic response rate.

#### 3.4.2 Safety outcomes

##### 3.4.2.1 Grade 3 or higher treatment-related adverse events

**Figure 4:**
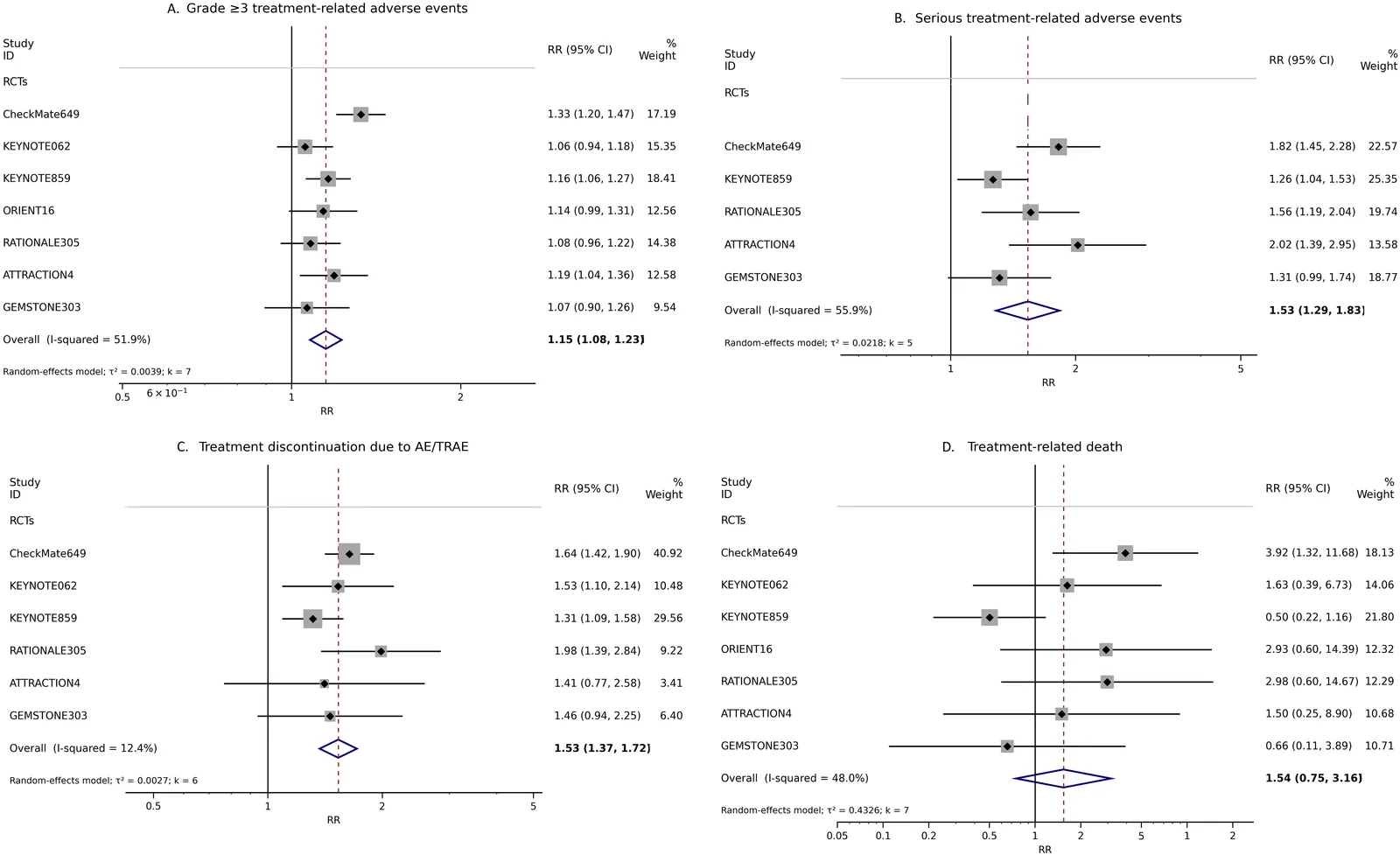
Forest plots of pooled safety outcomes. Forest plots comparing first-line PD-1/PD-L1 inhibitor plus chemotherapy with chemotherapy alone or placebo plus chemotherapy for **(A)** grade ≥3 treatment-related adverse events, **(B)** serious treatment-related adverse events, **(C)** treatment discontinuation due to adverse events or treatment-related adverse events, and **(D)** treatment-related death.

Data from all seven studies were incorporated into the analysis of grade 3 or higher treatment-related adverse events. **Figure 4A** displays the forest plot. The incorporation of a PD-1/PD-L1 inhibitor into chemotherapy correlated with a heightened incidence of treatment-related adverse events of grade 3 or higher, in contrast to chemotherapy alone or placebo combined with chemotherapy. The pooled risk ratio was 1.15 (95% CI, 1.08–1.23). Heterogeneity was moderate (I^2^ = 51.9%). A higher risk of severe treatment-related toxicity was expected with the addition of immune checkpoint inhibition to cytotoxic chemotherapy. The moderate heterogeneity is most likely due to differences in the chemotherapy backbones, treatment duration, reporting definitions, geographic populations, and immune checkpoint inhibitor agents. Although the incidence of serious treatment-related adverse events was higher, the safety profile of the combination regimens was generally reported to be manageable and consistent with the known toxicities of the individual treatment components in single trials.

##### 3.4.2.2 Serious treatment-related adverse events

For serious treatment-related adverse events, data were contributed from five trials. Forest plot is shown in **Figure 4B**. There was moderate heterogeneity (I² = 55.9%). The rise in serious treatment-related adverse events supports the need for careful patient selection, toxicity monitoring and early management of immune-related and chemotherapy-related toxicities. This safety signal should be interpreted in the context of the consistent improvement in OS and PFS.

##### 3.4.2.3 Treatment discontinuation due to AEs or TRAEs

Data on treatment discontinuation due to AEs or TRAEs were available from six trials. **Figure 4C** shows the forest plot. The exact attribution of discontinuation was not fully uniform across trials, with studies reporting discontinuation due to adverse events or treatment-related adverse events according to their individual trial definitions. Using these trial-reported definitions, treatment discontinuation was more common with PD-1/PD-L1 inhibitor plus chemotherapy than with chemotherapy alone or placebo plus chemotherapy (RR, 1.53; 95% CI, 1.37–1.72), with low observed statistical heterogeneity (I² = 12.4%). The pooled estimate should therefore be interpreted as reflecting a consistent increase in toxicity-related treatment discontinuation across closely related, but not identical, endpoint definitions.

##### 3.4.2.4 Treatment-related death

All seven trials contributed data to the treatment-related death analysis. The forest plot is shown in **Figure 4D**. The pooled RR was 1.54 (95% CI, 0.75–3.16), with moderate observed heterogeneity (I² = 48.0%). Although the pooled effect did not reach statistical significance, the estimate was substantially imprecise. The confidence interval ranged from a possible 25% reduction to a possible 216% increase in treatment-related mortality and therefore could not exclude clinically important harm or benefit. Accordingly, these findings should not be interpreted as demonstrating equivalence or confirming the absence of an effect on treatment-related mortality.

### 3.5 Subgroup analysis by trial region

Regional subgroup analyses compared trials conducted in Asia only or China only to global trials. The global trials were CheckMate 649, KEYNOTE-859, KEYNOTE-062, and RATIONALE-305. The Asian-only or Chinese-only trials were ATTRACTION-4, ORIENT-16, and GEMSTONE-303. **Table 3** summarizes the results.

**Table 3:** Subgroup analysis by trial region.

Outcome	Global trials			Asian/China-only trials			P for subgroup difference	Interpretation
	Effect estimate	95% CI	k	Effect estimate	95% CI	k		
Overall survival	HR 0.79	0.75–0.85	4	HR 0.81	0.73–0.91	3	0.70	OS benefit was observed in both trial settings, with no evidence of a subgroup difference.
Progression-free survival	HR 0.78	0.73–0.84	4	HR 0.66	0.58–0.74	3	0.02	PFS benefit was observed in both trial settings. The estimated effect was greater in Asian/China-only trials, although this exploratory trial-level difference should not be interpreted as patient-level regional effect modification.
Objective response rate	RR 1.25	1.16–1.34	3	RR 1.23	1.14–1.34	3	0.77	Response benefit was similar across trial settings, with no evidence of a subgroup difference.
Grade ≥3 treatment-related adverse events	RR 1.16	1.05–1.28	4	RR 1.14	1.05–1.24	3	0.79	No evidence of a subgroup difference was identified.
Serious treatment-related adverse events	RR 1.52	1.21–1.90	3	RR 1.60	1.05–2.44	2	0.83	No evidence of a subgroup difference was identified.
Treatment discontinuation due to AEs/TRAEs	RR 1.55	1.33–1.82	4	RR 1.44	1.01–2.05	2	0.71	Treatment discontinuation was increased in both regions.
Treatment-related death	RR 1.64	0.55–4.87	4	RR 1.50	0.56–4.03	3	0.91	Regional estimates were imprecise, and no evidence of a subgroup difference was identified.

AE, adverse event; CI, confidence interval; HR, hazard ratio; k, number of trials; OS, overall survival; PFS, progression-free survival; RR, risk ratio; TRAE, treatment-related adverse event. Subgroup-difference P values were calculated using Cochran’s Q test. Analyses were based on trial setting and should be considered exploratory.

The pooled HR for overall survival was 0.79 (95% CI, 0.75–0.85) in global trials and 0.81 (95% CI, 0.73–0.91) in Asian-only or China-only trials, with no evidence of a subgroup difference (P for subgroup difference = 0.70). The point estimates indicated negligible observed heterogeneity within both subgroups, although these estimates were based on only three or four trials. For progression-free survival, the pooled HR was 0.78 (95% CI, 0.73–0.84) in global trials and 0.66 (95% CI, 0.58–0.74) in Asian-only or China-only trials. The formal test indicated a difference between trial settings (P for subgroup difference = 0.02). However, this finding was based on only seven trials and should be considered exploratory and hypothesis-generating rather than evidence of definitive regional effect modification. The pooled RR for objective response rate was 1.25 (95% CI, 1.16–1.34) in global trials and 1.23 (95% CI, 1.14–1.34) in Asian-only or China-only trials, with no evidence of a subgroup difference (P for subgroup difference = 0.77).

Combination therapy was associated with higher risks of grade ≥3 treatment-related adverse events, serious treatment-related adverse events, and treatment discontinuation in both trial settings. Formal tests identified no evidence of subgroup differences for grade ≥3 treatment-related adverse events (P = 0.79), serious treatment-related adverse events (P = 0.83), or treatment discontinuation (P = 0.71). Treatment-related mortality estimates remained statistically inconclusive and imprecise within both subgroups, with no evidence of a subgroup difference (P = 0.91).

### 3.6 Sensitivity analyses

**Figure 5:**
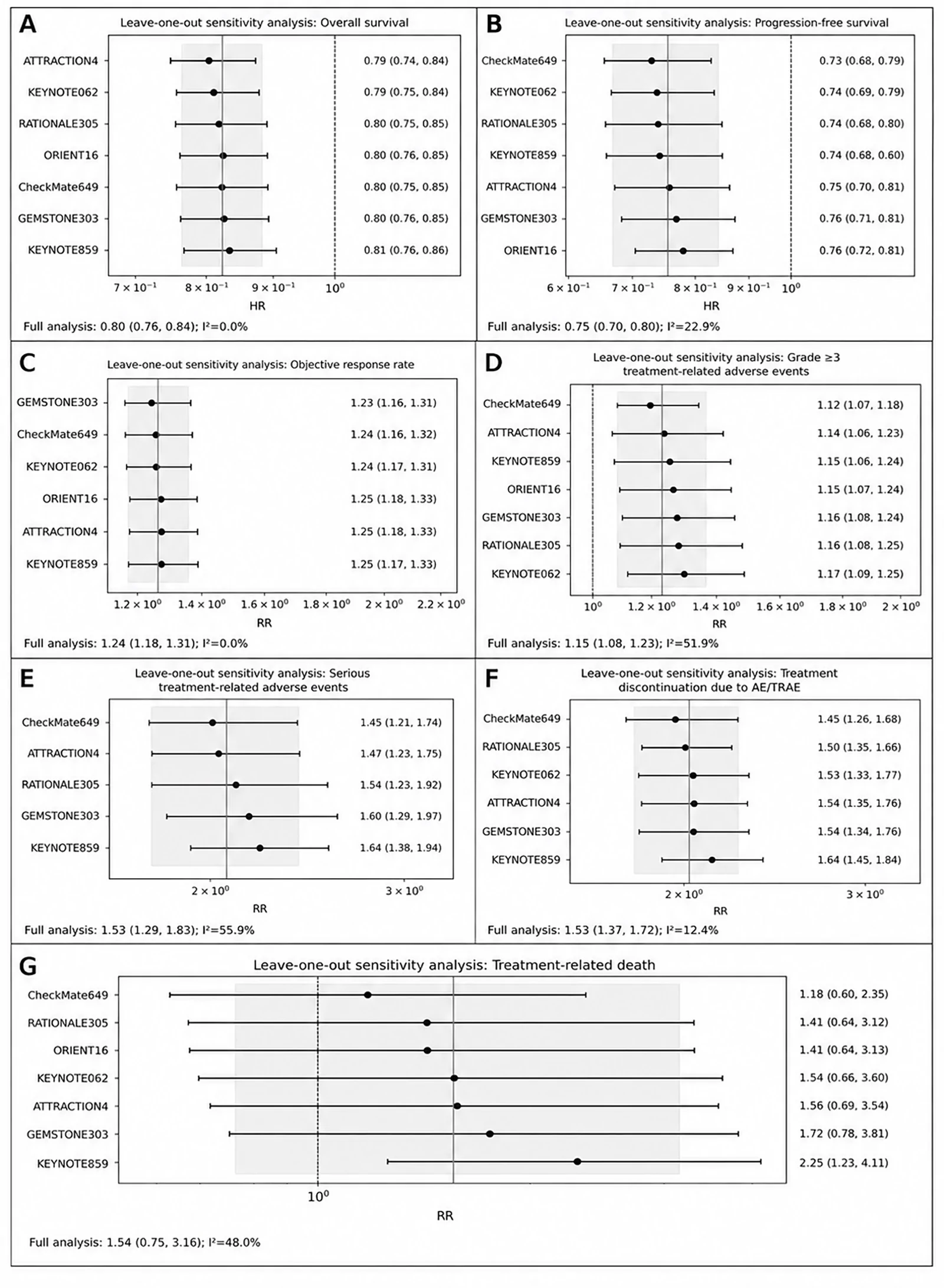
Leave-one-out sensitivity analyses. Leave-one-out sensitivity analyses evaluating the influence of each individual trial on the pooled estimates for **(A)** overall survival, **(B)** progression-free survival, **(C)** objective response rate, **(D)** grade ≥3 treatment-related adverse events, **(E)** serious treatment-related adverse events, **(F)** treatment discontinuation due to adverse events or treatment-related adverse events, and **(G)** treatment-related death. Each row represents the pooled effect estimate after excluding the indicated trial. The stability of the pooled estimates supports the robustness of the main efficacy and safety findings.

Sensitivity analyses were conducted by excluding one trial at a time and re-estimating the pooled effect estimate. Leave-one-out sensitivity analyses are presented in **Figures 5A–G**. Exclusion of each individual trial yielded directionally consistent pooled estimates of overall survival, progression-free survival, objective response rate, treatment-related adverse events of grade 3 or higher, serious treatment-related adverse events and treatment discontinuation. The results indicate that the primary efficacy and safety conclusions were not driven by any single study. In leave-one-out analyses, the pooled estimates for treatment-related death remained statistically inconclusive and imprecise. Exclusion of individual trials did not resolve the uncertainty surrounding this rare outcome, and clinically important increases or decreases in treatment-related mortality could not be excluded.

The fixed-effect and Hartung–Knapp-adjusted random-effects sensitivity analyses yielded effect estimates in the same direction as the primary DerSimonian–Laird random-effects analyses. Although the Hartung–Knapp adjustment produced wider confidence intervals for some outcomes, it did not materially alter the statistical interpretation or the principal efficacy and safety conclusions. Sensitivity analyses that included clinically relevant variations supported the robustness of the main results. Because KEYNOTE-062 and ATTRACTION-4 had neutral overall survival findings, leave-one-out analyses excluding each trial were clinically informative; neither exclusion materially changed the direction or magnitude of the pooled overall survival benefit. Clinically, the exclusion of GEMSTONE-303 was relevant as it was the only anti-PD-L1 trial and was limited to patients with a PD-L1 CPS of at least 5. Across the considerations, the direction of primary survival findings favored PD-1/PD-L1 inhibitor plus chemotherapy. .

### 3.7 Publication bias/small-study effects

**Figure 6:**
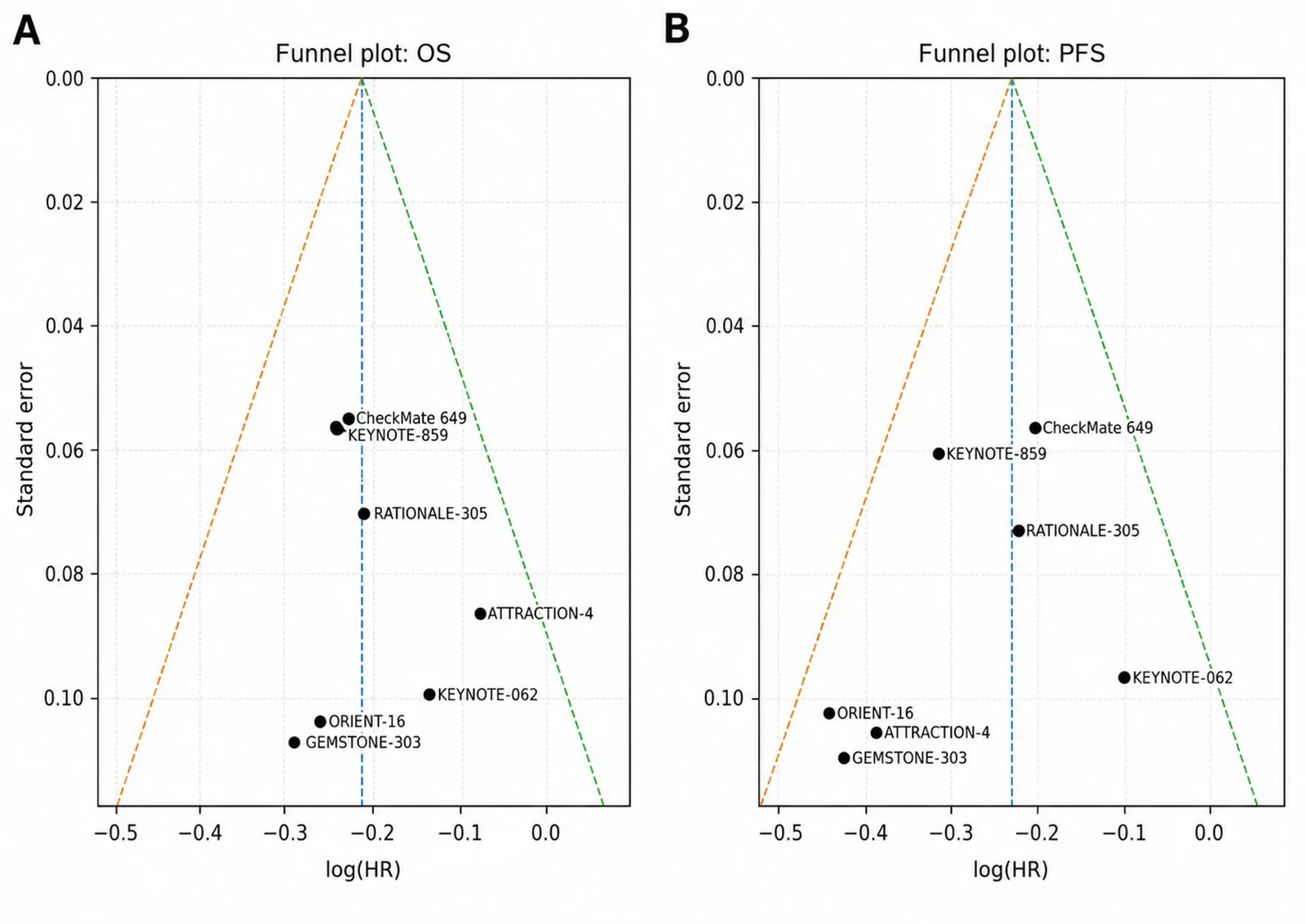
Funnel plots for publication bias and small-study effects. Funnel plots assessing potential publication bias or small-study effects for **(A)** overall survival and **(B)** progression-free survival. Each point represents an included randomized controlled trial.

Funnel plots were created for overall survival and progression-free survival to visually evaluate potential publication bias or small-study effects (**Figure 6**). No clear asymmetry was observed on visual inspection. Because only seven trials contributed to each analysis, formal tests of funnel-plot asymmetry were not performed.

### 3.8 Certainty of evidence

According to the GRADE framework, the certainty of evidence was rated as high for overall survival and progression-free survival. Although the included trials differed in geographic setting, biomarker eligibility, chemotherapy backbone, checkpoint inhibitor, and some design characteristics, these differences were not considered sufficient to warrant downgrading for indirectness because the trials directly addressed the same clinical question of first-line PD-1/PD-L1 inhibitor plus chemotherapy versus chemotherapy alone or placebo plus chemotherapy in advanced gastric or gastro-esophageal junction cancer. Clinical heterogeneity also did not translate into important statistical inconsistency: the direction of effect was consistent across studies, with negligible observed heterogeneity for overall survival (I² = 0.0%) and low observed heterogeneity for progression-free survival (I² = 22.9%).

No trial was judged to have a high overall risk of bias. Three trials had domain-specific concerns, but these were not considered sufficiently serious to downgrade the survival outcomes because the pooled effects remained directionally consistent and stable in leave-one-out sensitivity analyses. The pooled confidence intervals for both overall survival and progression-free survival were narrow and excluded the null, providing no basis for downgrading for imprecision. Although formal tests for publication bias were not performed because only seven trials were available, visual inspection of the funnel plots did not identify clear asymmetry. Taken together, these considerations supported retaining high certainty for the two primary survival outcomes. The certainty of evidence for objective response rate was rated as moderate because RATIONALE-305 lacked sufficient extractable arm-level response data, response-assessment methods varied across studies, and some risk-of-bias concerns were present in individual trials.

The certainty of evidence was rated as moderate for grade ≥3 treatment-related adverse events, serious treatment-related adverse events, and treatment discontinuation because of between-study heterogeneity, differences in safety definitions and reporting, and the limited number of contributing studies for some outcomes. The certainty of evidence for treatment-related death was rated as low because events were rare and the confidence interval was wide, resulting in substantial imprecision. Summary of certainty-of-evidence judgments are provided in **Table 2**.

## 4 Discussion

This systematic review and meta-analysis of seven randomized controlled trials involving 6,517 patients with advanced gastric or gastro-esophageal junction cancer demonstrated that first-line PD-1/PD-L1 inhibitors combined with chemotherapy significantly enhanced overall survival, progression-free survival, and objective response rate in comparison to chemotherapy alone or placebo plus chemotherapy. The magnitude and consistency of benefit were clinically meaningful: the pooled HR was 0.80 for overall survival, with negligible observed heterogeneity, and 0.75 for progression-free survival, with low observed heterogeneity. Nevertheless, heterogeneity estimates should be interpreted cautiously because only seven trials were available. These findings support first-line chemoimmunotherapy as a major therapeutic advance for advanced gastric and gastro-esophageal junction cancer, but also demonstrate a clear toxicity trade-off, with increased grade 3 or higher treatment-related adverse events, serious treatment-related adverse events and treatment discontinuation.

In the present analysis, we build on evidence from key phase 3 trials with a more precise and contemporary estimate of benefit for PD-1 and PD-L1 inhibitors. In CheckMate 649, nivolumab plus chemotherapy improved overall survival and progression-free survival, with sustained benefit confirmed after 5 years of follow-up. At a minimum follow-up of 60.1 months, survival benefits remained evident in the all-randomized population and across the reported PD-L1 CPS thresholds, with a greater magnitude of benefit at higher CPS cut-offs. These mature findings are consistent with the direction and durability of the treatment effect observed in our pooled analysis (7, 9). KEYNOTE-859 also demonstrated survival benefits of pembrolizumab plus chemotherapy in HER2-negative disease, further establishing the class-level benefit of PD-1 blockade with platinum-fluoropyrimidine chemotherapy (8). RATIONALE-305 added to this evidence base by demonstrating benefit with tislelizumab plus chemotherapy, indicating the chemoimmunotherapy effect is not confined to nivolumab or pembrolizumab (11).

Our results also align with those from Asian and China-only trials but highlight important regional nuances. ORIENT-16 demonstrated a survival benefit with sintilimab plus chemotherapy in a Chinese population and GEMSTONE-303 demonstrated a survival benefit with sugemalimab plus chemotherapy in PD-L1 CPS ≥5 disease, providing important evidence for PD-L1 inhibition in this setting (13, 17). The neutral overall survival result of ATTRACTION-4 should be interpreted in the context of its positive progression-free survival finding and East Asian trial setting. Although nivolumab plus chemotherapy improved progression-free survival, a statistically significant overall survival benefit was not observed, including on extended follow-up (14, 19). One possible explanation is that post-progression treatment and access to subsequent anticancer therapies may have reduced separation between the randomized groups for overall survival, despite improved disease control during first-line treatment. Regional treatment patterns and other trial-design features may also have contributed to the divergence between progression-free and overall survival. Therefore, ATTRACTION-4 does not necessarily contradict the efficacy of first-line chemoimmunotherapy; rather, it illustrates that progression-free survival and overall survival may diverge when subsequent treatment substantially influences long-term outcomes. Overall survival estimates were similar across trial settings, whereas the progression-free survival estimate was greater in Asian-only or China-only trials, with evidence of a trial-level subgroup difference (P = 0.02). Potential explanations include differences in chemotherapy backbones, subsequent treatment and access to immune checkpoint inhibitors after progression, enrolled patient populations, biomarker eligibility criteria, follow-up duration, and trial design. However, this finding should be considered exploratory because each subgroup contained only three or four trials and the classification was based on trial setting rather than individual patient geography or ethnicity. Global trials also included patients from Asian regions. Therefore, the observed difference may reflect ecological confounding and should not be used to guide region-specific treatment decisions.

KEYNOTE-062 also warrants separate consideration because pembrolizumab plus chemotherapy did not demonstrate superiority over chemotherapy for overall survival in the PD-L1 CPS ≥1 population (20). Differences from later positive trials may partly relate to its broader biomarker threshold, statistical testing framework, chemotherapy backbone, and treatment-effect heterogeneity across PD-L1-defined populations. In contrast, subsequent phase 3 trials, including CheckMate 649, KEYNOTE-859, ORIENT-16, RATIONALE-305, and GEMSTONE-303, demonstrated more consistent survival benefits with PD-1/PD-L1 blockade plus chemotherapy. The KEYNOTE-062 result should therefore not be disregarded, but should be interpreted as evidence that the magnitude of benefit is influenced by patient selection, biomarker definition, treatment regimen, and trial design. Importantly, exclusion of KEYNOTE-062 or ATTRACTION-4 in leave-one-out analyses did not materially alter the pooled overall survival estimate, indicating that the principal survival conclusion was not dependent on either trial.

The pooled findings should be interpreted in the context of substantial variation in biomarker eligibility and analysis populations. The included trials did not evaluate a single uniform PD-L1-defined population. KEYNOTE-062 prospectively enrolled patients with CPS ≥1, whereas GEMSTONE-303 was restricted to patients with CPS ≥5. Other trials enrolled broader or all-comer populations and reported additional analyses according to CPS or, in the case of RATIONALE-305, tumor area positivity. These scoring systems, thresholds, assays, and prespecified statistical populations are not directly interchangeable. To preserve independent randomized comparisons, the primary meta-analysis used intention-to-treat or all-randomized estimates when available. Biomarker-enriched subgroup estimates from all-comer trials were not pooled with their corresponding intention-to-treat estimates, because doing so would have included overlapping patient populations and assigned unequal weight to individual trials. However, when a trial enrolled only a biomarker-selected population, its randomized eligible population necessarily contributed to the pooled estimate. Consequently, the overall treatment effect represents an average across trials with different biomarker-selection strategies rather than a treatment effect applicable to a single PD-L1 threshold. The pooled results therefore support the overall efficacy of first-line chemoimmunotherapy but should not be interpreted as demonstrating an identical magnitude of benefit across CPS-low, CPS-high, TAP-defined, and biomarker-unselected populations. A trial-level comparison between biomarker-selected and all-comer studies was not performed because only two trials restricted enrollment by PD-L1 expression, their CPS thresholds differed, and between-trial comparisons would be susceptible to ecological bias and confounding by chemotherapy regimen, geographic setting, checkpoint inhibitor, follow-up, and trial design. Individual-patient-data meta-analysis using harmonized biomarker definitions would be required to determine reliably whether treatment effects differ according to PD-L1 expression.

The current findings are also consistent with previous meta-analyses but add important value by including newer trials and mature follow-up. Liu et al. (2022) showed that the combination of PD-1/PD-L1 inhibitors and chemotherapy improved outcomes but increased adverse reactions in untreated advanced gastro-esophageal adenocarcinoma. Likewise, Beshr et al. (2024) found that PD-1/PD-L1 inhibitors improved overall survival, and combination therapy was generally more effective than monotherapy (16). Lin et al. reported first-line immune checkpoint inhibitors plus chemotherapy improved efficacy with increased toxicity (15). Tong et al. (2025) focused on longer-term outcomes with PD-1 inhibitors plus chemotherapy in HER2-negative advanced gastric cancer (18). Unlike these analyses, our study provides an updated synthesis that includes GEMSTONE-303, mature CheckMate 649 and ATTRACTION-4 data, regional subgroup analyses, leave-one-out sensitivity analyses, RoB 2 assessment, and GRADE certainty ratings.

Results must be analyzed within the broader context of the initial treatment landscape. In HER2-positive disease, ToGA established trastuzumab combined with chemotherapy as a fundamental targeted strategy, while KEYNOTE-811 demonstrated that the addition of pembrolizumab to trastuzumab and chemotherapy enhances outcomes in HER2-positive metastatic gastro-esophageal cancer (19, 20). These studies are not directly comparable with our analysis because HER2-positive regimens were excluded when both arms were treated with anti-HER2 therapy, but they reinforce the principle that molecularly selected intensification of chemotherapy can improve survival. Furthermore, the SPOTLIGHT and GLOW studies showed that zolbetuximab plus chemotherapy was associated with improved outcomes in CLDN18.2-positive, HER2-negative gastric or gastro-esophageal junction adenocarcinoma (14, 21). These studies, collectively, imply that first-line treatment is increasingly biomarker-defined, with PD-L1, HER2, CLDN18.2, and MSI status directing the choice of therapy.

Later-line trials provide more perspective on the clinical significance of first-line benefit. Nivolumab activity in heavily pretreated Asian patients was delineated in ATTRACTION-2, but the absolute benefit in refractory disease was modest compared with the first-line chemoimmunotherapy effect seen in our analysis (22). KEYNOTE-061 did not exhibit a statistically significant advantage in overall survival with pembrolizumab compared to paclitaxel in previously treated PD-L1 positive illness, nor did JAVELIN. Gastric 100 did not exhibit enhanced overall survival with avelumab maintenance after first-line chemotherapy (23, 24). JAVELIN Gastric 300 also failed to improve survival with avelumab compared to chemotherapy in the third-line setting (25). The negative or modest later-line results further support the rationale for earlier use of immune checkpoint blockade in the context of potentially more favorable therapeutic settings, such as tumor burden, host immunity and chemotherapy-induced antigen release.

The safety findings are of clinical significance. The efficacy signal was strong, but combination therapy was associated with an increase in grade 3 or higher treatment-related adverse events, serious treatment-related adverse events, and discontinuation. The rise in discontinuation is especially important in gastric cancer, where patients often have poor nutritional reserve, anemia, peritoneal disease or impaired performance status. The absence of a statistically significant increase in treatment-related death should be interpreted with caution because of the rarity of events and the wide confidence intervals. These results are consistent with health-related quality-of-life analyses from CheckMate 649, which suggested that nivolumab plus chemotherapy might help preserve quality of life in selected patients, but underscore the importance of proactive toxicity management, early identification of immune-related adverse events, and a personalized approach to treatment duration.

These findings also reinforce the need for biomarker-integrated clinical decision-making. PD-L1 expression may enrich for benefit but remains an imperfect standalone predictor, and the clinical value of chemoimmunotherapy may also be influenced by microsatellite instability, Epstein–Barr virus status, HER2 expression, CLDN18.2 expression, tumor mutational burden, and features of the tumor immune microenvironment. Future trials should use harmonized biomarker assays and prospectively defined analysis populations to identify patients most likely to achieve meaningful benefit while avoiding unnecessary toxicity.

The study has a number of strengths. We only included randomized controlled trials, used mature data sets where possible, assessed efficacy and clinically relevant safety outcomes, used RoB 2 and GRADE frameworks, and evaluated robustness with leave-one-out sensitivity analyses and regional subgroup analyses. High certainty of evidence for overall survival and progression-free survival increases confidence in the primary conclusions. The negligible observed heterogeneity in the overall survival analysis is notable given the variation in checkpoint-inhibitor agents, geographic settings, and biomarker strategies, although uncertainty around the heterogeneity estimate remains because of the limited number of trials.

There are a couple of limitations to consider. First, this was an aggregate-data meta-analysis, which precluded patient-level evaluation by PD-L1 CPS, MSI status, ECOG performance status, age, disease burden, tumor site, race or ethnicity, and subsequent therapy. Second, the pooled analysis combined trial-level estimates from studies with different biomarker eligibility strategies, including all-comer populations and prospectively selected CPS-defined populations. Variation in PD-L1 assays, CPS and TAP scoring systems, thresholds, and statistical analysis populations prevented reliable direct comparison of biomarker-defined treatment effects. Because individual-patient data were unavailable, the analysis could not determine whether the magnitude of benefit differed according to PD-L1 expression, and the overall pooled estimate should not be assumed to apply uniformly across biomarker subgroups. Third, RATIONALE-305 could not be included in the pooled objective response rate analysis because sufficient arm-level responder data were unavailable and could not be reliably reconstructed from the reported information. Although the remaining six trials produced a precise pooled estimate, omission of one eligible trial may have resulted in either overestimation or underestimation of the overall response effect. Fourth, trials varied in chemotherapy backbone, immune checkpoint inhibitor agent, blinding, follow-up duration, and safety-outcome definitions; in particular, treatment discontinuation was variably attributed to AEs or TRAEs, which may have limited complete comparability of this pooled safety endpoint. Fifth, three trials were judged to have some risk-of-bias concerns, particularly in relation to deviations from intended interventions or selection of the reported result. Although no trial was classified as having a high risk of bias and the primary findings remained stable in sensitivity analyses, these judgments should be considered when interpreting the certainty and generalizability of the pooled estimates. Sixth, only one trial assessed a PD-L1 inhibitor, limiting the ability to compare PD-1 blockade with PD-L1 blockade. Seventh, some safety outcomes were based on a small number of contributing studies and treatment-related death was rare and imprecisely estimated. Finally, the interpretation of the funnel plot was limited by the small number of trials and publication bias could not be excluded with certainty. In addition, the review was not prospectively registered. Although the eligibility criteria, outcomes, and analytical framework were established before data extraction and quantitative synthesis.

## 5 Conclusion

This systematic review and meta-analysis, encompassing 7 randomized controlled trials with 6,517 patients suffering from advanced gastric or gastro-esophageal junction cancer, revealed a significant enhancement in overall survival, progression-free survival, and objective response rate when utilizing first-line PD-1/PD-L1 inhibitors in conjunction with chemotherapy, as opposed to chemotherapy alone or placebo combined with chemotherapy. The overall survival benefit was consistent across trials, robust in sensitivity analyses and was supported by high certainty evidence. However, combination therapy was also associated with increased risks of grade 3 or higher treatment-related adverse events, serious treatment-related adverse events and treatment discontinuation, whereas the effect on treatment-related death remained statistically inconclusive and substantially imprecise, with clinically important increases or decreases in mortality risk not excluded. These findings support PD-1/PD-L1 inhibitor plus chemotherapy as an effective first-line strategy for appropriately selected patients with advanced gastric or gastro-esophageal junction cancer. However, because the pooled trials used heterogeneous biomarker eligibility criteria and analysis populations, the overall estimate should not be interpreted as indicating an identical magnitude of benefit across all PD-L1-defined subgroups. Clinical use should weigh survival benefit versus risk of toxicity and factor in patient fitness, comorbidities, treatment goals and biomarker status. Future studies should select patients optimally, with integrated biomarkers such as PD-L1 expression, MSI status, HER2 status, CLDN18.2 expression, and tumor-immune features to maximize benefit and minimize unnecessary toxicity.

## Data Availability

The raw data supporting the conclusions of this article will be made available by the authors, without undue reservation.

## Supplementary material

Supplementary Table 1 Detailed design, eligibility, treatment, and biomarker characteristics of the included randomized controlled trials.

Supplementary Table 2 Trial-reported overall survival and progression-free survival estimates across PD-L1-defined populations.
