# Supplementary material for: Survival benefit and toxicity trade-offs of first-line chemoimmunotherapy in advanced gastric and gastroesophageal junction cancer: a meta-analysis of randomized trials
Source: Front Oncol. 2026 Sep 9;16:1914121. doi: 10.3389/fonc.2026.1914121 (PMC13597271; doi:10.3389/fonc.2026.1914121)
Supplement: Supplementary file 1 [file Table1.docx]

**Supplementary Table S1. Detailed design, eligibility, treatment, and biomarker characteristics of the included randomized controlled trials**

| **Trial and reference** | **First author/year** | **Region** | **Study design** | **Eligible population** | **HER2 status** | **PD-L1 eligibility/analysis population** | **Experimental regimen** | **Comparator regimen** | **Sample size, experimental/control** | **Primary endpoint(s)** |
| --- | --- | --- | --- | --- | --- | --- | --- | --- | --- | --- |
| **CheckMate 649** | Janjigian et al., 2021; 5-year update, 2026 | Global | International, multicenter, randomized, open-label phase 3 trial | Treatment-naïve patients with unresectable advanced or metastatic gastric, gastroesophageal junction, or esophageal adenocarcinoma | HER2-negative | Enrolled regardless of PD-L1 expression; primary efficacy analysis conducted in patients with CPS ≥5 | Nivolumab plus XELOX or FOLFOX | XELOX or FOLFOX | 789/792 | OS and PFS by blinded independent central review in patients with CPS ≥5 |
| **KEYNOTE-062** | Shitara et al., 2020 | Global | Randomized phase 3 trial with controlled and partially blinded treatment allocation | Previously untreated patients with locally advanced unresectable or metastatic gastric or gastroesophageal junction cancer | Not specified as an eligibility criterion in the manuscript table | PD-L1 CPS ≥1 required; CPS ≥10 population also analyzed | Pembrolizumab plus cisplatin and fluorouracil or capecitabine | Placebo plus the same chemotherapy | 257/250 | OS and PFS in patients with CPS ≥1 and CPS ≥10 |
| **KEYNOTE-859** | Rha et al., 2023 | Global | Multicenter, randomized, double-blind, placebo-controlled phase 3 trial | Treatment-naïve patients with locally advanced or metastatic gastric or gastroesophageal junction adenocarcinoma | HER2-negative | All-comer population; outcomes analyzed in the intention-to-treat, CPS ≥1, and CPS ≥10 populations | Pembrolizumab plus FP/cisplatin or CAPOX | Placebo plus the same chemotherapy | 790/789 | OS |
| **ORIENT-16** | Xu et al., 2023 | China | Randomized, double-blind, placebo-controlled phase 3 trial | Previously untreated patients with unresectable locally advanced or metastatic gastric or gastroesophageal junction adenocarcinoma | Not specified as an eligibility criterion in the manuscript table | All-comer population; CPS ≥5 subgroup also analyzed | Sintilimab plus XELOX | Placebo plus XELOX | 327/323 | OS in all randomized patients and in the CPS ≥5 population |
| **RATIONALE-305** | Qiu et al., 2024 | Global | Randomized, double-blind, placebo-controlled phase 3 trial | Patients who had received no previous systemic therapy for locally advanced unresectable or metastatic gastric or gastroesophageal junction adenocarcinoma | HER2-negative | All-comer population; TAP ≥5% and all-randomized populations analyzed | Tislelizumab plus CAPOX or FP/cisplatin | Placebo plus the same chemotherapy | 501/496 | OS in the TAP ≥5% population and in all randomized patients |
| **ATTRACTION-4** | Kang et al., 2022; 3-year update, Boku et al., 2024 | East Asia | Multicenter, randomized, double-blind, placebo-controlled phase 2/3 trial | Previously untreated patients with unresectable advanced or recurrent gastric or gastroesophageal junction cancer | HER2-negative | Patients were enrolled regardless of PD-L1 expression | Nivolumab plus SOX or CAPOX | Placebo plus SOX or CAPOX | 362/362 | Centrally assessed PFS and OS |
| **GEMSTONE-303** | Zhang et al., 2025 | China | Randomized, double-blind, placebo-controlled phase 3 trial | Treatment-naïve patients with unresectable locally advanced or metastatic gastric or gastroesophageal junction adenocarcinoma | Not specified as an eligibility criterion in the manuscript table | PD-L1 CPS ≥5 required | Sugemalimab plus CAPOX | Placebo plus CAPOX | 241/238 | OS and investigator-assessed PFS |

**Abbreviations:** CAPOX, capecitabine plus oxaliplatin; CPS, combined positive score; FOLFOX, fluorouracil, leucovorin, and oxaliplatin; FP, fluoropyrimidine; HER2, human epidermal growth factor receptor 2; OS, overall survival; PD-L1, programmed death ligand 1; PFS, progression-free survival; SOX, S-1 plus oxaliplatin; TAP, tumor area positivity; XELOX, capecitabine plus oxaliplatin. Sample sizes are presented as experimental/control.

**Supplementary Table S2. Trial-reported overall survival and progression-free survival estimates across PD-L1-defined populations**

| **Trial** | **PD-L1 metric** | **PD-L1-defined population** | **OS HR (95% CI)** | **PFS HR (95% CI)** | **Source/follow-up** |
| --- | --- | --- | --- | --- | --- |
| CheckMate 649 | CPS | ≥1 | 0.76 (0.67–0.85) | 0.77 (0.68–0.87) | 5-year follow-up |
| CheckMate 649 | CPS | ≥5 | 0.71 (0.61–0.81) | 0.71 (0.61–0.82) | 5-year follow-up |
| CheckMate 649 | CPS | ≥10 | 0.68 (0.58–0.79) | 0.68 (0.57–0.80) | 5-year follow-up |
| KEYNOTE-062 | CPS | ≥1 | 0.85 (0.70–1.03) | 0.84 (0.70–1.02) | Final analysis |
| KEYNOTE-062 | CPS | ≥10 | 0.85 (0.62–1.17) | 0.73 (0.53–1.00) | Final analysis |
| KEYNOTE-859 | CPS | ≥1 | 0.74 (0.65–0.84) | 0.72 (0.63–0.82) | Primary trial report |
| KEYNOTE-859 | CPS | ≥10 | 0.65 (0.53–0.79) | 0.62 (0.51–0.76) | Primary trial report |
| ORIENT-16 | CPS | ≥5 | 0.66 (0.50–0.86) | 0.63 (0.49–0.81) | Primary trial report |
| RATIONALE-305 | TAP | ≥5% | 0.71 (0.58–0.86) | 0.68 (0.56–0.83) | Updated/final analysis |
| ATTRACTION-4 | Tumor-cell PD-L1 expression | ≥1% | 1.11 (0.72–1.71) | 0.80 (0.50–1.27) | 3-year follow-up |
| ATTRACTION-4 | Tumor-cell PD-L1 expression | <1% or indeterminate | 0.83 (0.70–1.00) | 0.66 (0.53–0.82) | 3-year follow-up |
| GEMSTONE-303 | CPS | ≥5 | 0.75 (0.61–0.92) | 0.66 (0.54–0.81) | Final analysis |
| GEMSTONE-303 | CPS | ≥10 | 0.64 (0.48–0.85) | 0.58 (0.43–0.77) | Prespecified subgroup |

**Abbreviations:** CI, confidence interval; CPS, combined positive score; HR, hazard ratio; OS, overall survival; PD-L1, programmed death-ligand 1; PFS, progression-free survival; TAP, tumor area positivity. HRs <1 favor PD-1/PD-L1 inhibitor plus chemotherapy. Estimates are presented as reported by the individual trials and are descriptive only. PD-L1 assessment methods, scoring systems, thresholds, eligible populations, and follow-up durations differed across trials, and several biomarker-defined populations were nested or overlapping. Accordingly, these estimates should not be compared quantitatively across trials and were not pooled.
